# Internet Parent-Child Interaction Therapy for Maternal Guilt in a Child Abuse Case: A Single Case Study

**DOI:** 10.7759/cureus.70904

**Published:** 2024-10-05

**Authors:** Ayako Furuzawa, Naoki Yoshinaga, Kie Hattori

**Affiliations:** 1 Nursing, Nihon Fukushi University, Tokai, JPN; 2 Nursing, University of Miyazaki, Miyazaki, JPN

**Keywords:** child abuse, internet-based intervention, maternal guilt, parent-child interaction therapy, single-case study

## Abstract

This single case study explored the effectiveness of Internet Parent-Child Interaction Therapy (I-PCIT) for addressing maternal guilt in a case of child abuse. I-PCIT was implemented because traditional PCIT was challenging due to the family’s geographical constraints and the mother’s professional commitments. The study utilized multiple assessment tools, including the Eyberg Child Behavior Inventory (ECBI) and the Beck Depression Inventory-II (BDI-II), to measure the therapy’s impact on the child’s behavior and the mother’s depressive symptoms. The case conceptualization highlighted the mother’s emotional regulation difficulties and dissociative symptoms under stress that contributed to the abusive incidents. I-PCIT sessions, conducted via videoconferencing software, focused on enhancing the mother-son relationship through Child-Directed Interaction (CDI) and Parent-Directed Interaction (PDI) phases. Through 19 sessions of I-PCIT (with one session conducted face-to-face), the mother’s mastery of positive skills improved, and follow-up sessions indicated sustained positive outcomes. This case study underscores the potential of I-PCIT in preventing the recurrence of abuse, enhancing parental skills, and facilitating positive parent-child interaction. It also highlights the importance of therapist-parent collaboration in mitigating dropout risks and promoting therapy adherence.

## Introduction

Parent-Child Interaction Therapy (PCIT) [1] is widely considered to be an effective, evidence-based treatment for supporting families with children between the ages of two and seven years who are experiencing behavioral, emotional, and familial problems [2]. PCIT is a program grounded in behavioral, social learning, systems, and attachment theories, consisting of two phases: Child-Directed Interaction (CDI) and Parent-Directed Interaction (PDI). PCIT not only supports typical parent-child relationships but also has been shown in several clinical trials to prevent the recurrence of child abuse among at-risk families [3]. A previous randomized controlled trial found significant reductions in abuse reports among parents who completed PCIT compared to a control group [4]. The results suggest that PCIT can be a valuable intervention for preventing future instances of child abuse in families with a history of physical abuse.

In recent years, technological innovations, such as internet-based mental health care, have enhanced the importance of mental health care by providing treatment at home and alleviating some of the stigma associated with seeking professional help. The innovation of internet-based PCIT has also been rapidly delivered directly to families at home [5]. Furthermore, an Internet-PCIT treatment program (I-PCIT) may be well-suited for addressing certain types of maltreatment cases. The first controlled trial providing evidence for using real-time I-PCIT to deliver behavioral parent training remotely showed that 70% of children treated with I-PCIT demonstrated a treatment response post-treatment, with 55% maintaining this response at a six-month follow-up [6]. The effects on children’s symptoms and parental burden were similar to standard clinic-based PCIT, with I-PCIT showing higher rates of “excellent response” and fewer barriers to participation [6]. In 2022, following rapid developments in computer technology, mobile internet penetration in the United States was reported at 85% [7]. In the same year, in Japan, household ownership of information and communication devices, such as mobile phones, exceeded 90%, and the internet usage rate was 84.9% [8]. These facts also make internet-based parenting support a potentially important tool.

While PCIT has been proven effective in cases of child abuse, some high recidivism rates of physical abuse have been reported even after the PCIT intervention [4]. Parents who physically abuse their children often believe their actions are necessary for discipline rather than abuse [9]. Batzer et al. [10] reported high dropout rates from PCIT, particularly among families at risk for maltreatment, with an aggregated estimate of 48% attrition. PCIT requires mastery of skills in each phase before moving to the next, and early dropout can prevent caregivers from mastering these skills, thereby limiting the therapy’s effectiveness.

Even when a parent’s skills demonstrate improvement during clinical sessions, there can be challenges in reducing abusive behavior in their home environment. One suggested reason for this is that PCIT may not be universally effective for all cases of physical abuse, with cultural differences potentially influencing its implementation. Therefore, challenges remain in addressing physical abuse among parents and children, including high rates of disengagement, low parental motivation, distorted perceptions of child cognition that lead to physical violence, biases, and difficulties in generalizing skills within the home environment [11,12]. 

Globally, nearly three in four children (300 million) aged 2-4 years experience physical punishment and/or psychological violence at the hands of parents and caregivers [13]. The number of child abuse consultation cases reached 219,170 in 2022, an increase from 6,932 in 1998 [14]. Of the types of child abuse reported, psychological abuse was the most common, with a total of 219,170 cases (59.1%), followed by physical abuse, which accounted for 51,679 cases (23.6%). The perpetrators of abuse are often biological mothers [14], with one of the underlying factors being emotions associated with maternal guilt. Regarding the relationship between maternal physical abuse and guilt, mothers who physically abuse their children often experience significant guilt due to societal expectations of nurturing motherhood [15]. Maternal guilt can lead to emotional distress but can also motivate mothers to seek help and change their behavior. These findings underscore the importance of supportive interventions that address both the abusive behavior and the associated guilt. 

In the present case, the nursery reported an incident to the Child Guidance Center about the possibility of physical abuse. A distinctive feature of this case was that the mother adopted a coping mechanism of avoiding interactions with her son due to feelings of guilt about her parenting. The mother’s behavior pattern was to avoid playing with her child; however, she participated in I-PCIT, which resulted in a renewed relationship with her son. The relationship between the PCIT therapists and the mother was highlighted in the documentation of the process, particularly the mother’s feelings of wanting to drop out of I-PCIT. After several face-to-face interviews with the parent and child, it was noted that there was a considerable distance between their home and the PCIT room. To ensure they could attend sessions without feeling burdened and to prevent dropout, I-PCIT was implemented. However, the first session of the PDI was conducted in person, with the family coming to the session room, due to concerns about the reenactment of the trauma through the inclusion of a discipline scene.

## Case presentation

Background information

Ao (pseudonym) is a five-year-old boy who attends a nursery school. His mother, Sachiko (pseudonym), is 37 years old and is a full-time healthcare professional in charge of visiting rehabilitation. She works with children and adults and knows children’s growth and development and adult mental health. Ao’s father, who is 38 years old, is an engineer and works for an automotive company. The father waited in a separate room during each session, caring for Ao’s siblings. He was very cooperative in looking after the children. The mother mentioned that, at times, because of his involvement in caring for the children, he would be strict with their behavior. Ao’s parents also have an older daughter, aged seven years, and a younger daughter, aged two years.

Maternal grandparents lived an hour’s drive away and provided support when needed, but childcare and housework were primarily managed within the family, with most responsibilities handled by Sachiko.

Presenting complaints

This case involves Sachiko, who abused Ao and received warnings from nurseries on three occasions: First, when Ao was four years and four months old, she hit him in the face and locked him out of the front door; second, she grabbed Ao’s neck and caused him harm; third, she forced him to eat a rice ball and hit Ao when he threw it on the floor. After the third report, Ao was temporarily placed at his grandparents’ home and subsequently returned to his home. However, when Sachiko’s stress level increased, Ao was sent to his grandparents’ home even though there was no abuse. In this manner, the Child Guidance Center regularly met with and supported Sachiko from May 2022 to September 2023.

A staff member from the Child Guidance Center introduced PCIT to the parents; PCIT is one of the parent-training services used to improve the relationship between parents and children. Sachiko expressed interest and agreed to try PCIT. At the time of the referral, Sachiko was under increasing stress due to Ao’s repeated lies, sneaking sweets, and his rebellious and silent attitude when she reprimanded him. Sachiko wanted to learn from PCIT how she and Ao could manage their respective emotions.

History

Ao was born via normal delivery with a birth weight of 2,712 g. He did not experience any physical illnesses, injuries, or developmental delays. Ao began attending the nursery shortly after birth, where his elder sister, who was two years old, also went. The nursery teacher reported that Ao was well-behaved at the nursery, and a developmental test conducted at the Child Guidance Center did not identify any problems. Ao’s mother, Sachiko, had not received psychiatric consultations. Sachiko’s grandmother provided support for her and her children.

Assessment

The following questionnaires were used to assess Sachiko at the initial assessment: the Eyberg Child Behavior Inventory (ECBI) [16], the Beck Depression Inventory-II (BDI-II) [17], and the Dyadic Parent-Child Interaction Coding System (DPICS) [18]. Both the ECBI and BDI-II were also conducted before each subsequent therapy session.

The ECBI, which consists of 36 items, is administered to parents to assess behavioral problems in children between the ages of two and 16 years [16]. It features two scales: the Intensity scale and the Problem scale. The Intensity scale gauges how often disruptive behaviors occur, using a seven-point scale (1 = “never” to 7 = “always”) (clinical cutoff is ≥ 132; PCIT graduation criterion is ≥ 114). The Problem scale determines if parents consider these behaviors problematic, with responses marked as “yes” (1) or “no” (0) (clinical cutoff is ≥ 15). At the baseline assessment, Ao’s Intensity/Problem scores were 125/21. This indicated that Sachiko felt the difficulties of parenting were greater than the frequency of Ao’s problematic behaviors. The items on the ECBI indicating child behaviors that Sachiko found unpleasant or difficult included “dawdles in getting dressed,” “steals,” “lies,” “temper tantrums,” and “verbally and physically fights with sisters.”

The BDI-II comprises 21 items, each with a four-point response scale, and was used to measure Sachiko’s depressive symptoms [17]. At the baseline assessment, Sachiko scored 28, suggesting that she was experiencing moderate depression (clinical cutoff is ≥ 14).

The DPICS measures several categories of parent and child behaviors, which are documented using a video coding system based on frequency in real time [18]. We used the results of the DPICS to create a composite of “do” skills (behavior descriptions, reflections, and labeled praises) and “don’t” skills (questions, negative talk, and commands). In the baseline interaction between Sachiko and Ao, she tended to use more questions and indirect commands. 

Case conceptualization

Ao’s repetitive behaviors that were problematic for Sachiko (e.g., lying, sneaking food) were aimed at gaining her attention, which caused Sachiko to experience significant stress. It was not confirmed at the nursery whether these problematic behaviors were observed solely at home; Sachiko mentioned that these behaviors did not occur consistently. While managing parenting and household tasks in collaboration with her husband, Sachiko raised their three children, handled household chores, and balanced work responsibilities. As a result of this demanding situation, she also exhibited symptoms of depression.

Sachiko’s physical harm towards Ao, such as hitting him or injuring his neck, manifested from her difficulty with emotion regulation, leading to dissociative symptoms. Her physical abuse and the guidance received from the Child Guidance Center led to feelings of shame and was a deeply hurtful experience. The Child Guidance Center repeatedly emphasized to Sachiko the importance of avoiding any further instances of physical abuse. To prevent additional conflict with Ao, Sachiko adopted a coping strategy of avoiding one-on-one interactions and emotional engagement with him.

The therapists attempted to introduce PCIT to restructure their parent-child relationship to break the negative cycle occurring between them. PCIT was conducted by two therapists (KH and AF). The first author (AF) served as a co-therapist during the sessions and managed coordination with Sachiko and her family, as well as with administrative authorities. The focus was on acquiring PCIT skills, particularly spending time together, to gradually increase the amount of time Sachiko spent engaging with Ao. Additionally, the therapists aimed to build a relationship with Sachiko to facilitate her ability to express her emotions and improve her emotion regulation.

Course of treatment and assessment of progress

**Figure 1:**
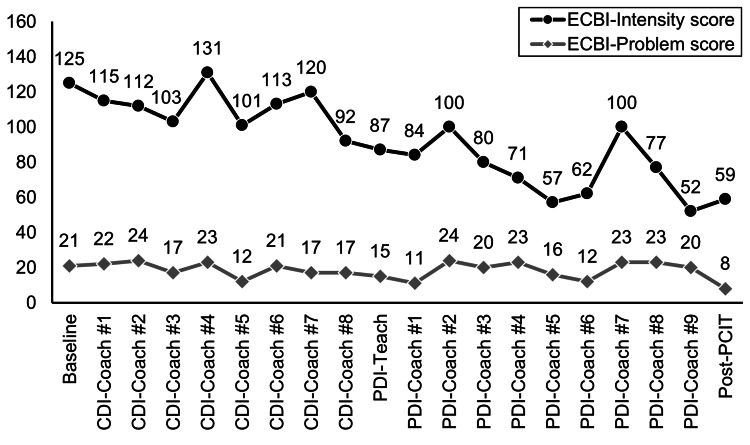
Mother’s ECBI scores over the course of I-PCIT CDI: Child-Directed Interaction; ECBI: Eyberg Child Behavior Inventory; I-PCIT: Internet Parent-Child Interaction Therapy; PDI: Parent-Directed Interaction

**Figure 2:**
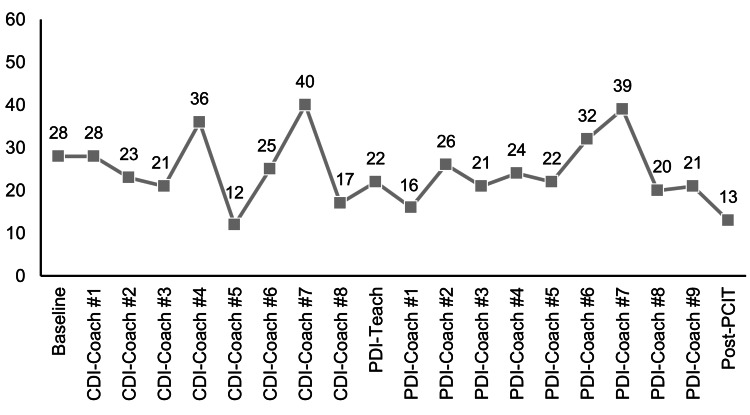
Mother’s BDI-II scores over the course of I-PCIT BDI-II: Beck Depression Inventory-II; CDI: Child-Directed Interaction; I-PCIT: Internet Parent-Child Interaction Therapy; PDI: Parent-Directed Interaction

Ao and his mother participated in weekly 60-minute sessions on the communications platform Zoom (Microsoft Corp., Redmond, WA) for a total of 18 sessions: eight sessions of CDI and 10 sessions of PDI, in addition to pre- and post-intervention sessions. The therapists determined that an online approach was the best option due to the family’s inconvenient residential location (an hour’s drive by car using the highway, in a remote area) and the parents’ additional responsibility for their two other children. However, due to Ao’s behavior, the first session of the PDI was conducted face-to-face. Sachiko’s scores over the course of I-PCIT are presented in Figure 1 for the ECBI and in Figure 2 for the BDI-II.

CDI Phase

The purpose of CDI sessions is to use PRIDE, an acronym that stands for Praise, Reflect, Imitate, Describe, and Enjoy, which teaches skills for building positive relationships with children and managing children’s inappropriate behavior. Some of these skills include ignoring negative behavior and praising positive behavior to reduce the occurrence of undesirable actions. The PRIDE skills in PCIT are fundamental techniques used to reinforce positive parent-child interactions and guide children’s behavior through positive reinforcement.

In the initial CDI teaching session (CDI-1), Sachiko followed Ao’s lead while he played, and employed three core “do” skills (i.e., labeled praise, reflection, and behavior descriptions) while using a learned skill to ignore his inappropriate behavior. In CDI-1, Ao and his mother did not play together. Ao exhibited attention-seeking behaviors by leaving the room - the session was conducted at his home via Zoom. Sachiko tried to convince Ao to return by calling out to him loudly. In CDI-3, Ao’s attention-seeking behaviors diminished, and Sachiko mentioned that she no longer felt anger.

CDI-4 was suddenly canceled on the scheduled session date because Sachiko’s colleague had passed away, and she had to cover the deceased colleague’s remaining work tasks. She also felt profound sadness, and her BDI-II score worsened. The therapists were concerned that Ao and Sachiko might withdraw from PCIT due to her stress and avoidant reactions. Therefore, the therapists maintained a consistent waiting stance and provided psychological support for Sachiko through email. The CDI-4 session was rescheduled for three weeks later. Characteristics of this session included the continued maintenance of Sachiko’s CDI skills and the development of an attachment relationship between Ao and his mother. Sachiko expressed fear about not completing this session, feeling frustrated with Ao, and not completing her homework. The therapists acknowledged Sachiko’s feelings, shortened the session, and allocated time for discussion with her. During the discussion, she stated, “I am glad I did the session today,” and mentioned feeling refreshed after receiving coaching. Since the CDI-4, Sachiko has been able to express her emotions and concerns to the therapists and did not cancel any sessions until the end of PCIT.

Continuing with the I-PCIT sessions was observed as a relationship repair, providing Ao and his mother with the necessary time to acquire and improve skills. Sachiko mentioned many opportunities to spend time with Ao and subsequently discussed her changing perspective. By CDI-8, Sachiko’s skills had reached mastery (defined as having 10 behavioral descriptions, reflections, and labeled praises coded within the five-minute observation, as determined by the DPICS coding). Regarding the change in the frequency of Ao’s behaviors, which were observed almost every day at baseline, Sachiko reported that “lying” was no longer observed after CDI-3, and “temper tantrums” were reduced by half after CDI-5; however, “verbal/physical fights with sisters” continued to be observed almost every day during the CDI phase. The ECBI-Intensity scores (representing the frequency of a child’s problematic behaviors) were gradually improved, from 125 to 95 throughout the CDI phase, with some temporal fluctuations.

PDI Phase

In the PDI phase, effective commands are modeled for and practiced by clients, and reinforced by the therapists. Mastery of PDI skills is defined as: (a) at least 75% of commands given by the parent are effective, and (b) 75% of the parent’s commands are followed correctly (i.e., with labeled praise for compliance and appropriate use of the time-out warning/procedures for noncompliance).

The first PDI coaching session (PDI-1) was conducted in person rather than via Zoom. PDI was conducted to address child discipline and was expected to be more emotionally challenging for the mother. It also required greater family cooperation than CDI and included observing the relationships between the father and siblings. The PDI procedures were thoroughly covered in the session, with all family members participating. The father provided indirect support, such as caring for Ao’s siblings while Ao and Sachiko played together. Sachiko was understood to be in charge of the PDI procedures; however, she often struggled to give clear instructions, resorting to indirect commands. This indicated a lack of confidence and a hesitancy to exercise leadership with her child, which possibly reflected her past abusive actions toward him. In the PDI sessions, the therapist took the lead in coaching to ensure consistent behavior and correct adherence to the time-out procedures.

In the PDI-7, Sachiko discussed with the therapists the commands she had given to Ao (as many as 10 times) that had proven to be ineffective. She felt frustrated and had not spoken with Ao since then. Sachiko explained that the excessive expectations she had of Ao (e.g., “he must be this way”) reflected her desire for him not to become an adult who cannot regulate his emotions, as she herself struggles to do. By the end of the PDI phase, Sachiko was able to communicate appropriate commands and had achieved mastery of the necessary skills. Regarding the change in the frequency of Ao’s behaviors during the PDI phase, Sachiko reported that the frequency of “verbal/physical fights with sisters” had reduced after initiating the PDI phase, and “temper tantrums” were rarely observed after PDI-4. At the post-PCIT assessment, scores on the ECBI-Intensity/Problem and BDI-II were well below the clinical cutoff.

Complicating factors

The Child Guidance Center faced several challenges because of limited personnel and budget. This made it difficult for them to handle numerous cases, and they suffered from a lack of resources for one-on-one attention in severe abuse cases, insufficient coordination with other institutions, and inadequate long-term follow-up for affected children [19]. Therefore, a case like the current one, in which the mother understands the situation, feels guilty about the physical abuse, and is troubled by her actions, might be considered relatively less severe. As a result, the response from the Child Guidance Center could have been delayed.

Difficulties in the PCIT process for Sachiko and Ao were primarily linked to Sachiko’s guilt, daily stressful events, and the relationship between her and the therapists. While working full-time, Sachiko took on a central role in childcare and household responsibilities for her three children. The stress she reported encompassed various factors beyond the children’s stress, including the loss of an individual for whom she was caring and her own physical well-being. Additionally, she felt deeply distressed about Ao’s concerning behaviors. Sachiko’s times of high stress were reflected in her BDI-II scores; during those periods, the frequency of her homework with Ao decreased, and opportunities for playing with him were reduced. Sachiko adopted avoidant behaviors to prevent potential abuse stemming from difficulties in emotional regulation during high-stress situations. There was a correlation between Sachiko’s BDI-II scores and Ao’s ECBI scores: when Sachiko’s BDI-II scores were high, Ao’s ECBI scores also tended to be elevated. By CDI-8, Ao’s ECBI scores had gradually decreased to below 100, and he responded to Sachiko’s PRIDE skills, leading to an increase in his positive behaviors. Thus, the change in Sachiko positively influenced her perspective of Ao.

Furthermore, there were risks of dropout, and Sachiko experienced emotional challenges around CDI-4 due to the loss of her colleague and her demanding work schedule as she managed her deceased colleague's remaining tasks. Sachiko was also experiencing depression and feelings of guilt about not being able to spend time with her children, including Ao. Indeed, dropout is a known issue in PCIT for parents and children at risk of abuse. While the therapists also grappled with potential problems, they endeavored to maintain their relationship with Sachiko. In this case, two therapists were involved, and mutual support between them was essential for their shared responsibility. The two therapists held a meeting outside of therapy sessions to share information. They assigned roles: the main therapist, who would conduct the sessions, and the assistant therapist, who would collaborate with other professionals and the family. To address the dropout risk and Sachiko’s emotional challenges while maintaining her relationship with the therapists, the therapists normalized her difficult situation, empathized with her, and informed her that she could temporarily take a break from the sessions (i.e., allowing for a postponement of the sessions). The therapists also provided positive feedback regarding Sachiko’s ability to use the CDI skills with Ao, even when she spent limited time with her children, by utilizing the PRIDE skills, which helped to enhance her sense of self-efficacy.

Access and barriers to care

The therapy was primarily conducted online. In cases involving abuse, safety, and risk management are paramount in I-PCIT. Therapists must establish protocols for addressing emergency safety concerns, ensure confidentiality, and build trust in an online setting. Therapists need to employ strategies to engage the family and foster trust and rapport. Having the support of family members as a backup system during sessions to respond to emergencies is also necessary.

Additionally, Sachiko’s coping mechanism of avoiding interactions with Ao was identified as a barrier to care, as it allowed her to evade feelings of shame and guilt associated with being notified by the Child Guidance Center about the alleged abuse of her child. Thus, the mother developed a negative cycle in her relationship with her child. However, by participating in PCIT sessions and establishing positive interaction with Ao through CDI, Sachiko eventually felt more at ease in her multifaceted role.

Follow-up

A follow-up online session with Sachiko was conducted three months post-PCIT. Sachiko’s ECBI Intensity/Problems scores (87/18) remained relatively stable. One year after Sachiko received notification about her child abuse, the following factors had positively changed: (i) Ao is doing well at the nursery, but at home, he is prone to tantrums when things do not go his way and when his demands are not met; however, while Sachiko remains concerned about these behaviors, she can now seek help from the nursery teacher; (ii) Sachiko’s emotions are stable; and (iii) she has been able to manage Ao and his siblings’ care more effectively.

## Discussion

Treatment implications of the case

The findings of this case study support the effectiveness of I-PCIT in preventing the recurrence of abuse. PCIT was primarily conducted online rather than face-to-face and demonstrated effectiveness in regulating the mother’s emotions and preventing the reenactment of her traumatic relationships with therapists. In response to feelings of guilt about her treatment of her child, Sachiko avoided one-on-one interactions with him to prevent further physical abuse. Consequently, the opportunity for her to build a warm relationship with her child was lost. Therefore, since trauma adversely affects relationships [20], avoiding the reenactment of a traumatic relationship with a therapist may help mitigate the negative effects that a client experiences from discontinuing I-PCIT.

Additionally, in response to Sachiko’s difficulties with emotion regulation and dissociative symptoms during the periods of increased stress, the therapists provided her with live coaching that focused on the present moment. We argue that this intervention (Coaching from the Ear) can prevent the occurrence of dissociative symptoms and support what is commonly known as grounding. For example, Sachiko reported that although the coaching was conducted online, hearing the live coaching made her “feel calmer” and enabled her “to interact more effectively with Ao.” Indeed, in vivo coaching of parents using behavioral principles has been shown to be a powerful mechanism of change in many behavioral parent-training programs [21-23]. Furthermore, Sachiko mentioned that “the therapist’s voice in my ear was very calming,” which may have helped her avoid dissociation and instead “be here now.” Shame and fear of being judged as parents are key barriers to seeking help [24]. In this case, Sachiko experienced shame and deep hurt on receiving guidance from the Child Protection Services regarding accusations of physical abuse. The relationship between a parent and professionals such as therapists or Child Guidance Center staff (including Child Protective Services) can easily become a traumatic experience.

The collaboration and support among the therapists were crucial in preventing the reenactment of Sachiko’s traumatic relationships. Our commitment to Sachiko involved consistently maintaining an attitude of working together whenever a challenge arose and engaging in collaborative discussions, which were essential skills in her PCIT treatment process. Furthermore, it is noteworthy that Sachiko’s experience of not reenacting a traumatic relationship with the therapists during the PCIT treatment process positively impacted her relationships with staff at the preschool and Child Protective Services, even after completing PCIT.

According to Lieneman et al. [25], home-based PCIT has been explored to address the high attrition rates seen in clinic-based models. Home-based PCIT offers benefits such as increased session frequency, skill generalization, and eliminating transportation barriers, although it also presents home-based distractions. Graziano et al. [26] found in their randomized trial that while I-PCIT showed significant effects on treatment outcomes, standard PCIT demonstrated better long-term maintenance of reduced externalizing behavior problems. However, I-PCIT achieved a low dropout rate of 3%, making it promising for high-stress families needing rapid improvements. Building and maintaining a strong therapeutic alliance can be challenging in an online setting [5]. However, conducting several face-to-face interviews with the parents and child before implementing I-PCIT helped to confirm and sustain motivation, leading to better outcomes. The success of I-PCIT relied heavily on active participation and engagement from both parents and children. Taking into account these considerations in I-PCIT and the measures taken in this case, the therapist must employ strategies to engage the family and foster trust and rapport with them, such as having a few in-person sessions within I-PCIT.

Recommendations to clinicians and students

In cases of abuse, ensuring safety and managing risk are crucial in I-PCIT. Therapists must establish emergency safety protocols, maintain confidentiality, and build trust within an online setting. Flexible techniques and effective parental coaching are essential components of the therapy process. Supervision was provided in this case to ensure the proper implementation of PCIT. Although intervention from the Child Guidance Center had ended, periodic reports on the status of PCIT were provided to the agency. Emergency response protocols were confirmed, and multiple supervisions were conducted to ensure adherence to strict implementation protocols. Confidentiality was maintained, and consent was obtained from the mother.

It is also crucial to understand the underlying factors contributing to problematic behaviors and the associated parental responses, such as guilt toward their child. Each case presents unique challenges and circumstances; therefore, therapists must consider factors such as cultural background, socioeconomic status, and mental health. Emphasis should be placed on supporting parents in developing their emotion regulation skills and addressing their trauma-related issues. Techniques such as live coaching and grounding experiences can be valuable tools in helping parents manage stress and prevent dissociation. Furthermore, establishing a trusting and supportive relationship between the therapist and the parent(s) is paramount. Therapists and students should prioritize building rapport, demonstrating empathy, and fostering an environment in which parents feel safe to express their concerns and challenges. The journey toward healing and positive change is ongoing. The support described in this study can help continuously reinforce positive behaviors and prevent relapse in many parent-child therapy contexts.

## Conclusions

This single case study utilized I-PCIT to address maternal guilt associated with child abuse. It underscores the potential of I-PCIT in preventing the recurrence of abuse, enhancing parental skills, and facilitating positive parent-child interactions. This case study also highlights the importance of collaboration between therapist and parent in mitigating dropout risks and promoting adherence to therapy.
